# Development of On-Line Monitoring Systems for High Temperature Components in Power Plants

**DOI:** 10.3390/s131115504

**Published:** 2013-11-13

**Authors:** Hongcai Zhang, Jiuhong Jia, Ning Wang, Xiaoyin Hu, Shan-Tung Tu, Shaoping Zhou, Zhengdong Wang

**Affiliations:** 1 Key Laboratory of Pressure Systems and Safety, Ministry of Education, East China University of Science and Technology, Shanghai 200237, China; E-Mails: nwang@ecust.edu.cn (N.W.); huxy@simt.com.cn (X.H.); sttu@ecust.edu.cn (S.-T.T.); shpzhou@ecust.edu.cn (S.Z.); zdwang@ecust.edu.cn (Z.W.); 2 SINOPEC Hubei Chemical Fertilizer Branch, Zhijiang 443200, China; E-Mail: hcaizhang@sohu.com

**Keywords:** on-line monitoring system, strain, extensometer, high temperature, accuracy

## Abstract

To accurately detect deformation and extend the component life beyond the original design limits, structural safety monitoring techniques have attracted considerable attention in the power and process industries for decades. In this paper an on-line monitoring system for high temperature pipes in a power plant is developed. The extension-based sensing devices are amounted on straight pipes, T-Joints and elbows of a main steam pipeline. During on-site monitoring for more than two years, most of the sensors worked reliably and steadily. However, the direct strain gauge could not work for long periods because of the high temperature environment. Moreover, it is found that the installation and connection of the extensometers can have a significant influence on the measurement results. The on-line monitoring system has a good alarming function which is demonstrated by detecting a steam leakage of the header.

## 1. Introduction

Many power plants commissioned in the 1960s are currently approaching the limits of their original design lives. Although there has been a great effort to find an optimum during design, to ensure the highest quality in the manufacturing process and especially to specify the loadings and the load-time histories of the systems and components, it is an accepted fact that some of the systems are subjected to “not-specified” loading in service. For instance, loadings can be caused by a change in operating parameters, by abnormal operation or by the fact that local effects are not known in detail. Any attempt to cover all these possible loadings in fatigue calculations is unrealistic. Therefore, monitoring the minute changes of the component level should be a desirable way to ensure the safety of these old plants. The on-line health monitoring involves the observation of a system using periodically sampled dynamic response measurements from an array of sensors, the extraction of damage-sensitive features from these measurements, and the statistical analysis of these features to determine the current state of system health. It can detect early degradation of material properties associated with operational usage, service loads, and environmental exposure, and predict future material properties at critical locations for components, structures and complex systems subjected to service loads and environmental exposure over time.

For major parts in power plants, changes of the temperature and the pressure are well correlated with the creep in most cases [1,2]. Thus, it is believed that the real-time strain is the most reliable parameter for component life monitoring in power plants [3].

The main challenges in monitoring high temperature strain come from the harsh working environment and extremely small deformations of components. In order to solve this problem, Riza and Sheikh proposed the concept of a new hybrid class of all-silicon carbide optical sensor for use under high temperature conditions [4], but their experiment was only conducted for 350 min, therefore its long time reliability still needs further proof. Tu *et al.* developed a local deformation measuring technique using optical fiber marking and remote monitoring for high temperature creep testing [5], which was rather difficult to use on-site. Morris *et al.* presented the ARCMAC strain measurement system using image analysis [6,7]. Notably, these methods are restricted by the resolution of the tele-microscope and image processing accuracy. Thereby, an extension-based sensing device (extensometer for short) was designed and verified both in the laboratory and on-site [8–10]. In this paper a monitoring system with extensometers for on-site high temperature components is introduced and its main features are discussed by practical application in a thermal power plant.

## 2. Selection of Monitoring Locations

**Figure 1.**
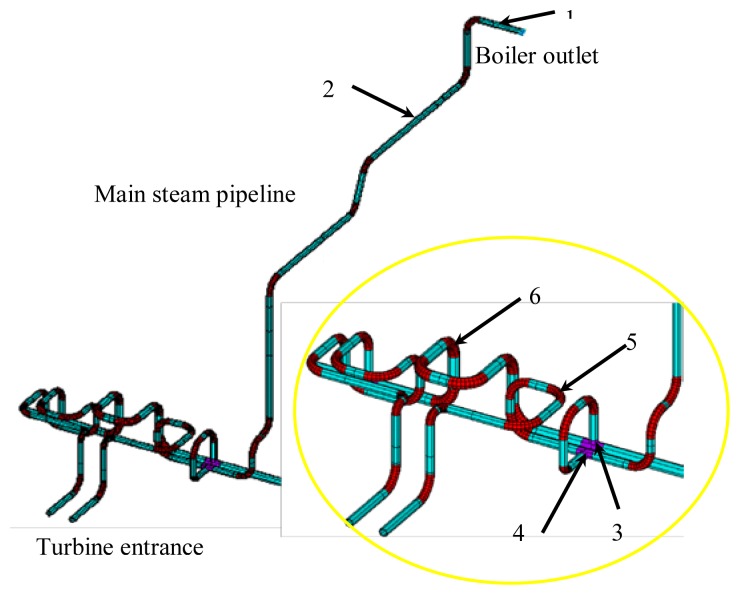
Diagram of the main steam pipeline and monitoring points.

Due to its importance and practical need, the present work focuses on the monitoring of a main steam pipeline. Both the finite element method and engineering experiences are chosen for analysis. During the numerical simulation, piping is selected as shown in Figure 1, which is a part of the main steam piping of a thermal power plant. The working temperature is 540 °C and the pressure is 9.8 MPa. Table 1 shows the material properties and the design parameters of the piping. The dimensions of the main steam piping are 273 mm in diameter, 28 mm in thickness and 1,370 mm in bend radius.

**Figure 2.**
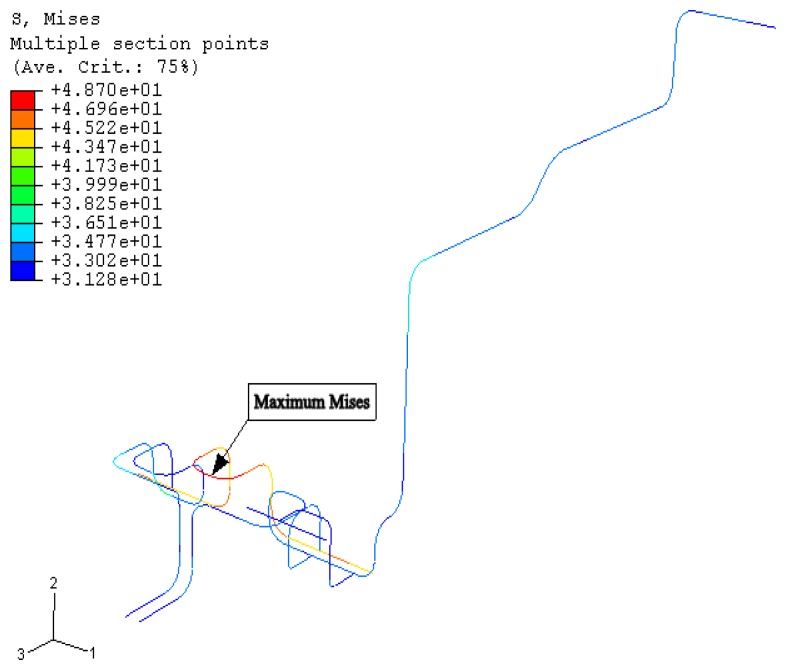
Von Mises stress contour of the piping system.

In order to distinguish the “weak” points on the piping system, a von Mises stress contour of the piping system was analyzed using finite element software as shown in Figure 2. The conclusion can be drawn that the maximum stress does not exceed the allowable stress under the working conditions. The higher stress part matches the given location of the design drawings, where the monitoring equipment needs to be installed. The other relatively “weak” parts are bends, and the T-joints. Combining analysis and engineering experiences, the monitoring points are selected as shown in Figure 1. Points 1 and 2 are at the straight pipe near the boiler header. Points 3 and 4 are at weld seams on the T-pipe, and points 5 and 6 were at elbows of the pipe.

## 3. Data Analysis of the Long-Term Monitoring System

### 3.1. Introduction of the Monitoring System

**Figure 3.**
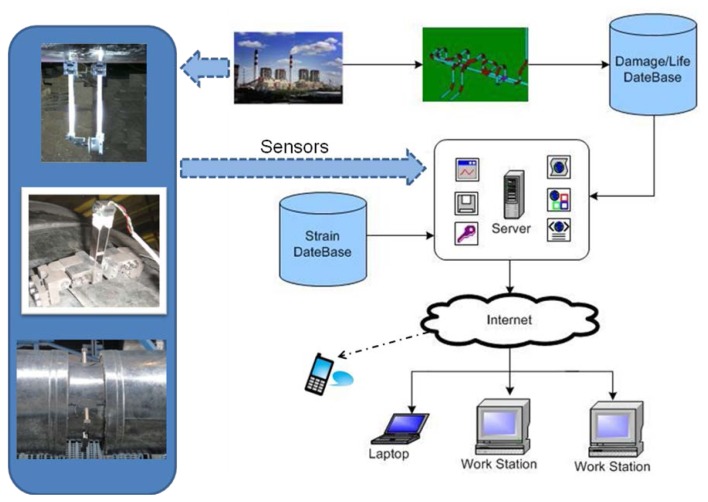
Modules of the monitoring system.

This system is designed to predict the life of high temperature piping according to on-line strain monitoring [11]. The configuration of its several modules is shown in Figure 3.

This system includes sensors, a strain data base, a finite element analysis module, a life prediction module and an alarming module. When the monitoring strain data is input into the system, simulation of life prediction of the “weak” pipe points is implemented by the finite element method. Relationships between the damage and the residual life are retrieved according to the database, and the remaining life of the piping is evaluated. If the input signal is abnormal, the system will alarm. All signals from the monitoring system and conversing data are collected and input into the corresponding database in the server, and it can be easily accessed by remote clients through the Internet.

### 3.2. Installation of the Extensometers

**Figure 4.**
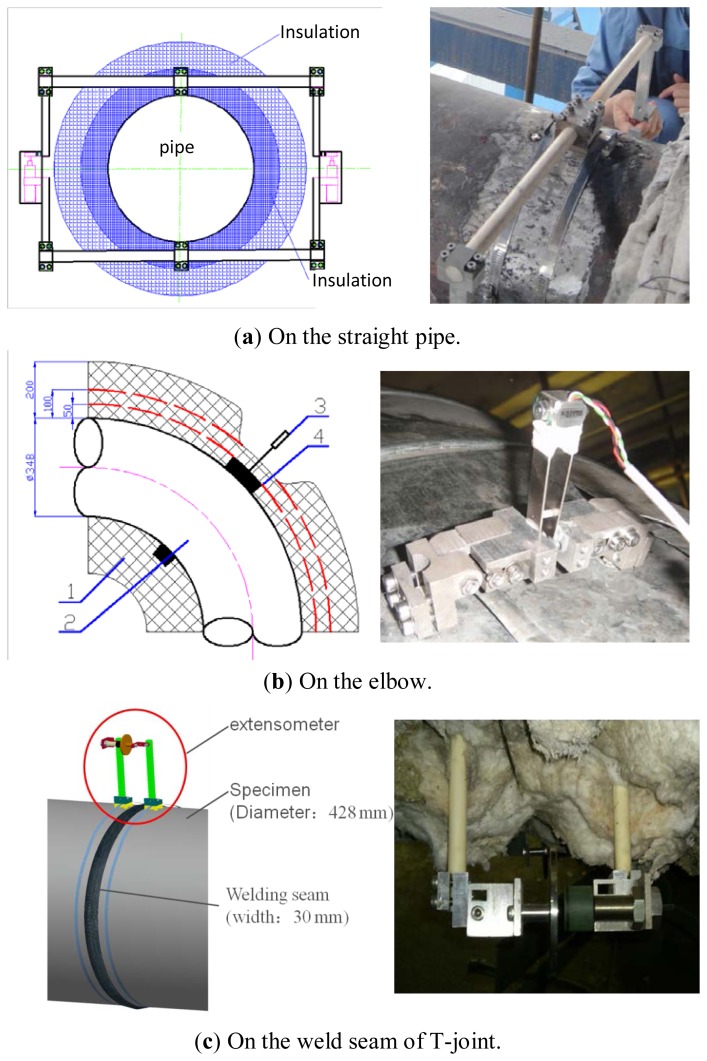
Installation of the extensometers.

According to locations selected above, the extensometers are installed. Detailed information about the installation of extensometers on stations 1 and 2 was presented in [8], as well as that of stations 3 and 4 in [9], and stations 5 and 6 in [10]. Pictures of the installation are shown in Figure 4. The reliability of these extensometers has been verified in the lab. However, in order to inspect their performance on site, some direct strain gauges have been installed at each station near the extensometers, and the monitoring data of the strain gauges acts as references. These strain gauges used on-site are KHCM-10-120-G15-11C2M units supplied by KYOWA (Tokyo, Japan).

### 3.3. Main Features of the Long-Term Monitoring System

#### 3.3.1. Longer Working Time of Extensometers than Direct Strain Gauges

**Figure 5.**
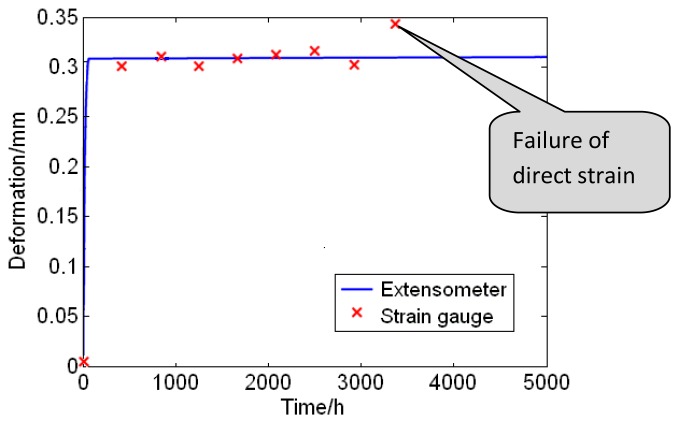
Deformation comparison of the extensometer and the strain gauge.

At the beginning, all the strain gauges worked well. However, one of the strain gauges stopped working one month later during the monitoring process. Gradually, other ones lost their effectiveness, and the longest working time was no more than 3,000 h. On the contrary, the extensometers can be stably used for about two years. This data can be found in Figure 5, which shows the strain monitoring information of the welding seam on a T-joint. In order to display a better comparison result, monitoring data of 5,000 h is selected. The curve is obtained by the minimal square error method. The strain rate calculated by the data of extensometer is a constant. However, the strain rate calculated by the strain gauge increases quickly at about 2,965 h and later become infinite. Moreover, the temperature and pressure of the main steam inside the piping are normal. Therefore, we can estimate that the strain gauge is damaged. Obviously the extensometers can be used longer than the strain gauges.

#### 3.3.2. Effect of Extensometer Installation

To monitor high temperature creep deformation of the pipe in the plant, extensometers can solve the problems of harsh working environment and extremely small deformation of components. However, there are some other difficulties. During the on-site tests, it was found that the fixation method is a factor that could not to be ignored.

**Figure 6.**
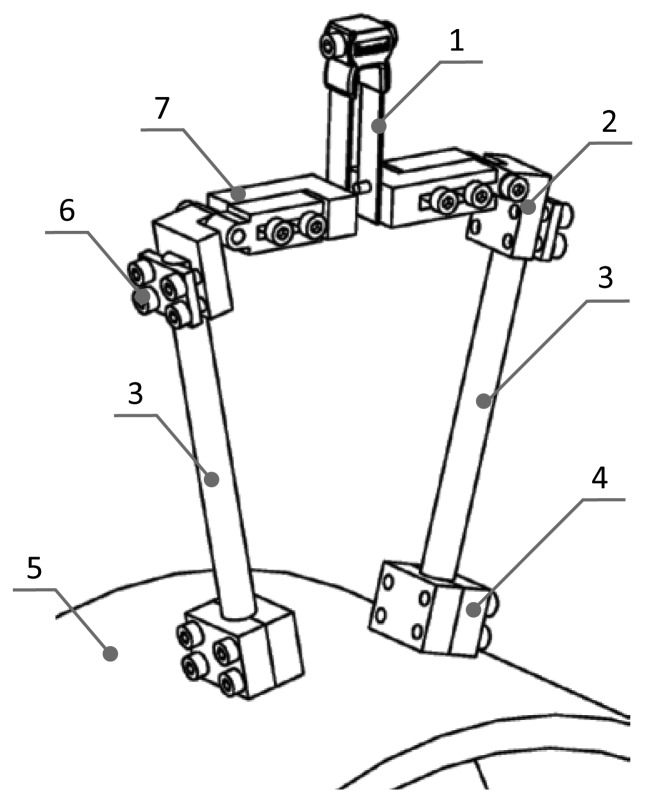
Structure of the extensometer. (**1**) Sensor; (**2**) Fixing mechanism; (**3**) Ceramic rod; (**4**) Position block; (**5**) Specimen; (**6**) Mounting bolt; (**7**) Connecting mechanism.

On a T-joint, there are two extensometers to monitor the same welding seam, and the structure of the extensometer is composed of a sensor, a fixing mechanism, a ceramic rod, position blocks, mounting bolts and a connecting mechanism. Through the position blocks, the extensometer is fixed on the specimen, as shown in Figure 6.

**Figure 7.**
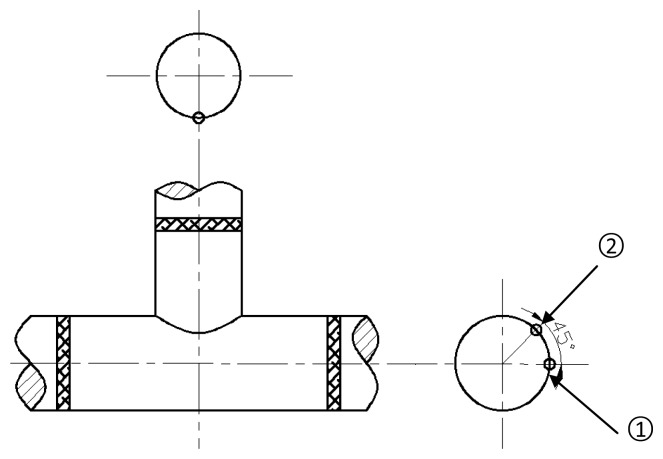
Location schematic of two extensomers on the same weld seam of a T-joint.

**Figure 8.**
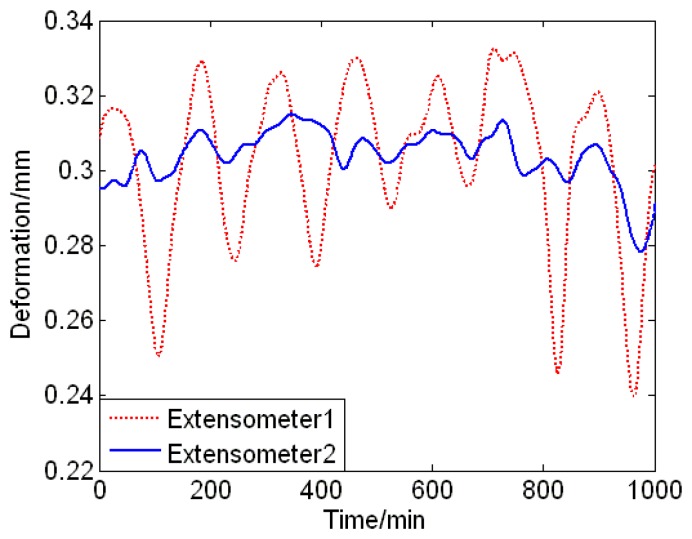
Original data from two extensometers with different fixation methods.

The detailed installation locations on T-joint are shown in Figure 6. The position blocks of extensometer 1 are banded by clamp, which is made of 10CrMo910. The position block of extensometer 2 is both banded by a clamp and welded to the T-joint. According to the original data, it is easy to find that the data sensed by two different extensometers differed greatly, as shown in Figure 7. The data of the extensometer 2 dovetails nicely with that of the strain gauge during its effective working period, which can be seen in Figure 8. Therefore, the conclusion can be drawn that the fixation method can have a significant influence on the measurement results.

#### 3.3.3. Alarming Function of the Monitoring System

When the monitoring system had been put into use for about three months, there was an alarm. The operators began to check the piping system at once. It was found that there was a leakage on the boiler header. Therefore, they stopped to repair it.

**Figure 9.**
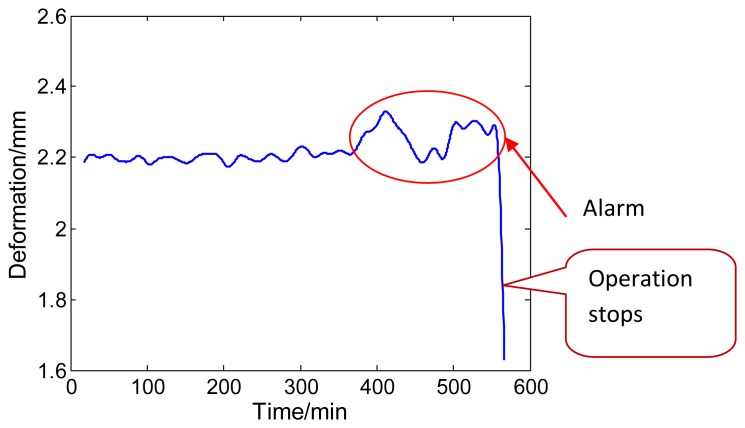
Deformation monitoring curve with alarm.

In order to analyze strain changes during alarming, deformation data of the straight pipe for about 560 min before leakage is selected, as shown in Figure 9. This data was recorded by the computer without further processing. According to this figure, it is easy to find that the measuring deformation data of pipe is not a constant. The pressure and the temperature of the steam flowing inside the pipe give rise to this fluctuation during the actual operation of the power plant. Based on fluctuation features of the pipe deformation curve, a rate of deformation of the pipe can be calculated by the least square fitting method. In this system the deformation rate is regarded as the creep rate. The appropriate deformation rate of the pipe can be input into the system's life prediction module in a timely fashion.

The system alarms when the deformation rate of the pipe is greater than the threshold value, which can be seen in Figure 9. Early warning of creep rate levels can prevent major catastrophes. This function greatly reduces the plant operation risks and operator workload.

## 4. Conclusions

In this paper, an on-line creep deformation monitoring system for high temperature pipes is introduced briefly, and its features are studied and summarized when put into use on the main steam piping in a thermal power plant. According to on-site long-term monitoring tests, it is found that extensometers can work much longer than direct strain gauges. However, much more attention should be paid to the fixation method of the extensometers in the harsh working environment, because the installation and connection will affect monitoring results. Moreover, the system alarm function can detect accidental risks, which has been proved by finding a water vapor leakage when used in the plant. Therefore, the on-line health monitoring system is a valuable method to detect deformation rate of high temperature pipelines and forecast plant risks.

## Figures and Tables

**Table 1.** Material properties and design parameters of the piping.

**Material**	**Density/kg·m^−3^**	**Modulus of Elasticity/GPa**		**Linear Expansion Coefficient/**°**C**	**Allowable Stress/MPa**	
	
		**20** °**C**	**540** °**C**		**20** °**C**	**540** °**C**
10CrMo910	7,800	214.4	173.1	1.4 × 10^−5^	150	52
